# Wave I_n_ in auditory brainstem response suggests a high possibility of a high jugular bulb

**DOI:** 10.3389/fped.2023.1183388

**Published:** 2023-11-06

**Authors:** Jia Liu, Wanqin Xie, Yan Ding, Ya Hu, Ruosha Lai, Peng Hu, Ganghua Zhu

**Affiliations:** ^1^ Department of Otolaryngology-Head and Neck Surgery, The Second Xiangya Hospital, Central South University, Changsha, Hunan, China; ^2^ National Health Commission Key Laboratory of Birth Defects for Research and Prevention, Hunan Provincial Maternal and Child Health Care Hospital, Changsha, China

**Keywords:** auditory brainstem response, high jugular bulb, hearing loss, cochlear implant, wave I

## Abstract

**Background:**

Wave I_n_, which refers to the negativity between waves I and II in auditory brainstem response (ABR), is an electrophysiological phenomenon observed in previous studies. The term “high jugular bulb” (HJB) describes a jugular bulb that is located in a high position in the posterior aspect of the internal acoustic canal. The present study aimed to explore the correlation between wave I_n_ and the possibility of a HJB.

**Methods:**

This retrospective study included a cohort of pediatric patients diagnosed with profound hearing loss who were enrolled in a government-sponsored cochlear implantation program at an academic medical center between January 2019 and December 2022. The analysis involved examining the results obtained from the ABR test and high-resolution computed tomography (HRCT) of the temporal bone in the patients. The position of the jugular bulb was classified according to the Manjila and Semaan classification.

**Results:**

A total of 221 pediatric patients were included in the study. Twenty-four patients, with a median age of 3 years and a range of 1–7 years, showed significant bilateral (*n* = 21) or unilateral (*n* = 3) wave I_n_ (mean latency: right ear, 2.16 ms ± 0.22 ms; left ear, 2.20 ms ± 0.22 ms). The remaining 197 patients showed an absence of ABR. The HRCT images revealed that 18 of the 24 patients (75%) had HJB, but only 41 of the 197 patients who lacked ABR (20.8%) showed signs of HJB. The ratio difference was considered statistically significant based on the chi-squared test (*χ*^2^ = 32.10, *p* < 0.01). More than 50% of the HJBs were categorized as type 4 jugular bulbs, which are located above the inferior margin of the internal auditory canal.

**Conclusion:**

ABR wave I_n_ in pediatric patients with profound hearing loss suggests a high possibility of HJB. The physiological mechanism underlying this correlation needs further investigation.

## Introduction

Auditory brainstem response (ABR) refers to the short-term neural electrical activity that is recorded from the scalp and originates from the inner ear, auditory nerve, and auditory brainstem under air- or bone-conducted acoustic stimulation (1). ABR has been commonly used to evaluate the integrity of the auditory pathway. A typical click-evoked ABR shows five primary waves labeled using Roman numerals Ⅰ–Ⅴ in sequence. Each ABR wave comprises a positive peak followed by a negative one, termed, e.g., P1 (or peak I) and N1 (or I_n)_ (2, 3). The threshold, amplitude, and latency of ABR waves are key parameters for clinical interpretation (4). In a previous study, Martin et al. (5) noted a negative correlation between waves I and II (wave I_n_) in ABR recorded from human patients undergoing neurosurgical procedures and considered that wave I_n_ could be stationary potentials originating from conductivity boundaries existing in the posterior fossa. In a subsequent independent study, Rattay and Danner (6) proposed that peaks I_n_ are stationary potentials when volleys of spikes cross the external electrical conductivity barrier at the interface between the bone and dura/cerebrospinal fluid, supporting Martin’s hypothesis.

A jugular bulb (JB) refers to a bulbous enlargement at the junction of the intracranial sigmoid sinus and internal jugular vein (7). The JB is located in the jugular fossa, and the position of the jugular fossa varies among different individuals. The term “high jugular bulb” (HJB) describes anatomical variants of the JB rising to the level of the basal turn of the cochlea, encroaching upon the floor of the internal auditory canal, or protruding into the tympanic cavity or inner ear (8). Reportedly, the incidence of HJB ranges from 6% to 20% in patients undergoing computed tomography (CT) of the temporal bone for any reason (8, 9). A recent retrospective study shows a prevailing rate of 42% for HJB (predominantly unilateral) among 194 children who underwent cranial CT primarily due to head trauma (10). Awareness of vascular abnormalities is beneficial in minimizing clinical complications during otologic surgery (11). Of note, a recent case–control study has demonstrated that HJB is associated with hearing loss in patients diagnosed with bilateral large vestibular aqueduct syndrome (LVAS) (14).

Based on the previous findings, we assumed that the presence of ABR wave I_n_ could be related to certain anatomical variants in the posterior fossa. Thus, we explored the correlation between wave I_n_ and HJB in a cohort of pediatric patients with profound hearing loss who were enrolled for cochlear implantation.

## Materials and methods

### Medical ethics

This retrospective study was approved by the Institutional Review Board (IRB) of the Second Xiangya Hospital of Central South University, and written informed consent was obtained.

### Patients

The patients included in the study were children with profound deafness who were enrolled for government-sponsored cochlear implantation at the Department of Otolaryngology of the Second Xiangya Hospital of Central South University between January 2019 and December 2022. The patients underwent ABR testing and high-resolution computed tomography (HRCT) of the temporal bone as part of their presurgical evaluation.

### ABR measurement and determination of wave I_n_

ABR testing was performed with a Neuro-Audio system (Neurosoft Ltd., Ivanovo, Russia) in an acoustically and electrically shielded booth. The active, reference, and ground electrodes were placed in the middle of the forehead at the hairline, the bilateral mastoids, and the nose root, respectively. The resistance between electrodes was ≤4 kΩ. An ER-3A plug-in earphone (Etymotic Research, Inc., Elk Grove Village, IL, USA) was used to deliver the click stimulus of 1,024 sweeps at a rate of 21.1 /s, with a bandpass filter setting from 100 Hz to 3,000 Hz and a recording time window of 15 ms. The stimulus intensity started from 80 dB nHL and declined/increased in 10 dB increments. The measurement was repeated at least three times for each stimulus level. A threshold of ≤30 dB nHL for click-ABR wave V was considered to be within the normal range (0 dB nHL = 28.7 dBSPL). The latency of wave I_n_ is in reference to the previously reported value (mean, 2.06 ms; standard deviation, 0.11 ms) (3).

#### HRCT

HRCT scans were performed on a Somaton Plus 4A CT scanner (Siemens AG). The acquisition parameters are as follows: 120 kVs, 100 mAs, 0.75 mm collimation, 1 mm reconstruction increment, a pitch factor of 1, and a field of view of 100 mm. All radiographs regarding the position of JB were reviewed by two radiologists unaware of the study design. Referencing the Manjila and Semaan classification, JB was classified as follows: type 1: no bulb; type 2: below the inferior margin of the posterior semicircular canal (PSCC); type 3: between the inferior margin of the PSCC and the inferior/margin of the internal auditory canal (IAC); and type 4: above the inferior margin of the IAC (11).

### Data analysis

Descriptive statistical analysis of the numerical data (including age and wave latency) and the chi-squared test on frequencies were processed by software Origin 8.0 (OriginLab Corporation, Northampton, MA, USA).

## Results

**Figure 1:**
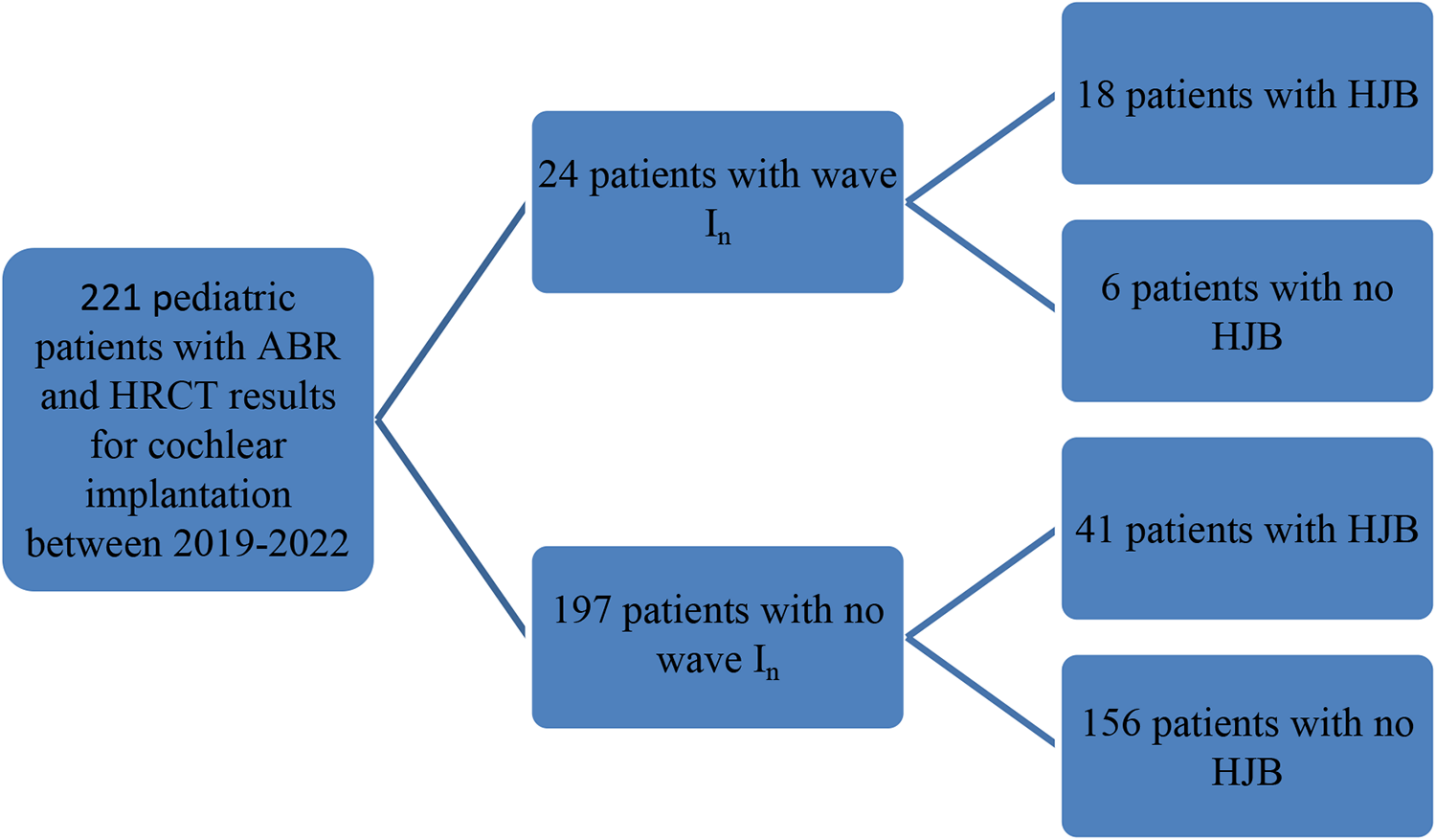
Flowchart showing the grouping of study participants.

**Figure 2:**
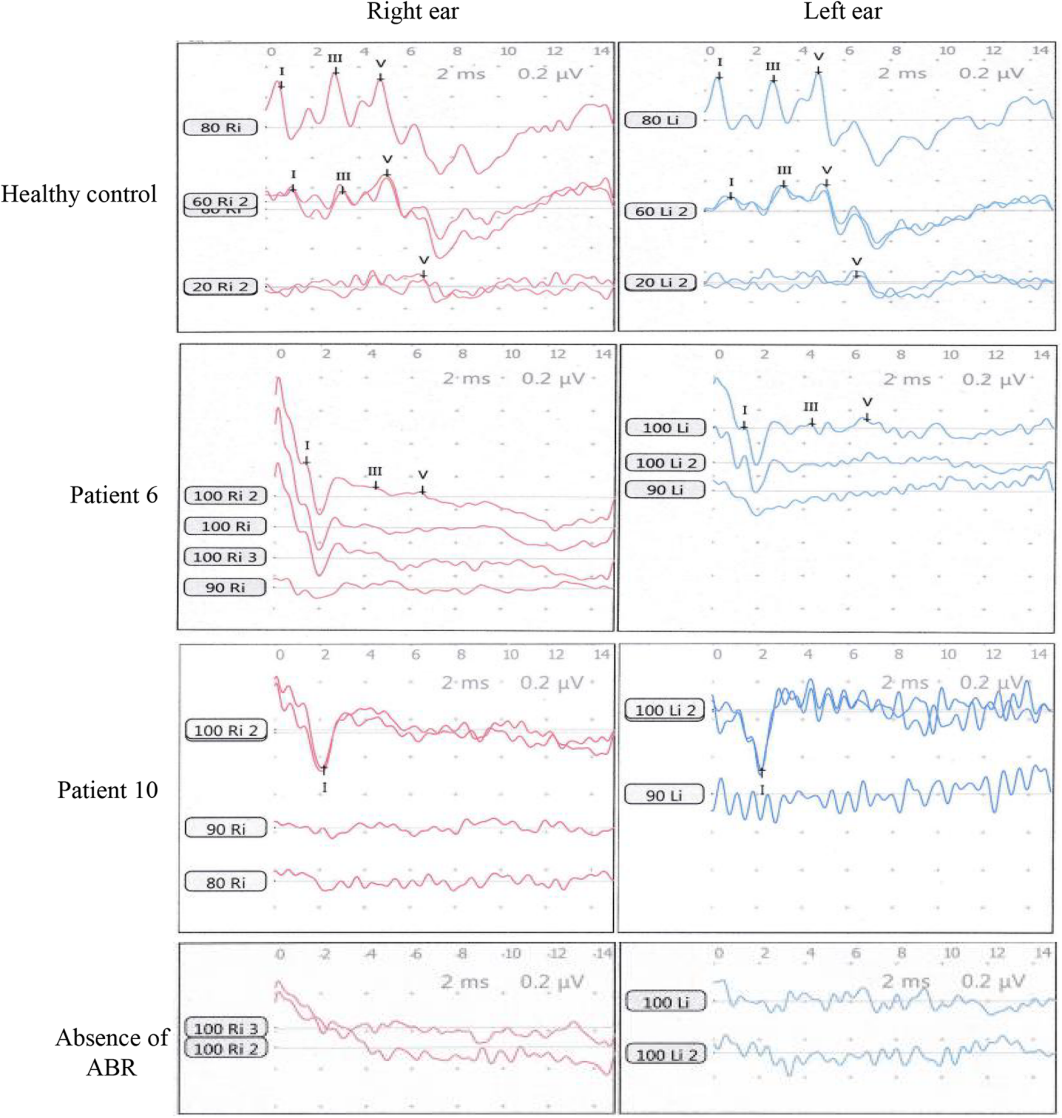
ABR waves I_n_ were recorded from the pediatric patients with profound hearing loss. The healthy control was a boy of age 2.5 years with normal hearing. ABR waves of patient 6 and patient 10 in Table 1 are representatively shown. A control of the absence of ABR was shown at the bottom panel.

A total of 221 pediatric patients were included in the study. There were 24 patients, comprising 14 boys and 10 girls, with a median age of 3 years and a range of 1–7 years, who had significant bilateral (*n* = 21) or unilateral (*n* = 3) wave I_n_ (mean latency: right ear, 2.16 ms ± 0.22 ms; left ear, 2.20 ms ± 0.22 ms). The other 197 patients showed an absence of ABR at the maximum stimulation intensity of 100 dB nHL (Figures 1 and 2 and Table 1). The HRCT images revealed that 18 of the 24 patients (75%) exhibited bilateral (*n* = 8) or unilateral (*n* = 10) HJB, compared with 41 of the 197 patients who lacked ABR (20.8%) and showed bilateral or unilateral HJB. The difference in ratios was considered statistically significant based on the chi-squared test (*χ*^2^ = 32.10, *p* < 0.01). According to the Manjila and Semaan classification of the JB location, 15 of the 26 (58%) HJBs in the patients with wave I_n_ belong to type 4 JBs and six (23%) and five (19%) HJBs belong to type 3 and type 2 JBs, respectively (Table 1).

**Table 1:** Demographics and clinical features of the patients with wave I_n_.

Patient No.	Age (years)	Sex	ABR	Right ear			Left ear			I_n_ latency
			R/l (dB nHL)	I_n_	HJB	Type	I_n_	HJB	Type	R/l (ms)
1	5	M	100/90	×	○	Ⅳ	×	○	Ⅳ	2.22/2.28
2	1	F	80/100	×	○	Ⅳ	×	○	Ⅱ	2.17/2.22
3	2	M	80/100	×	○	Ⅲ	×	○	Ⅳ	1.64/2.12
4	3	M	100/90	×	○	Ⅲ	×	○	Ⅱ	2.01/2.01
5	4	F	100/90	×	○	Ⅱ	×	○	Ⅱ	2.22/2.38
6	2	M	90/90	×	○	Ⅳ	×	○	Ⅳ	1.96/1.96
7	5	M	100/100	×	○	Ⅳ	×	○	Ⅳ	2.70/2.70
8	5	M	100/100	×	○	Ⅳ	×	○	Ⅳ	2.10/2.14
9	3	F	90/80	×	○	Ⅳ	×			2.28/2.54
10	1	F	100/100	×	○	Ⅳ	×			2.12/2.17
11	4	M	100/90	×	○	Ⅲ	×			2.59/2.33
12	2	F	100/100	×			×	○	Ⅳ	2.38/2.06
13	2	M	100/100	×			×	○	Ⅲ	2.06/1.96
14	1	F	100/100	×			×	○	Ⅲ	2.22/2.65
15	6	M	90/90	×			×	○	Ⅲ	1.96/1.85
16	1	M	100/100	×			×	○	Ⅳ	2.10/2.20
17	4	F	80/100				×	○	Ⅱ	NA/2.00
18	3	M	80/80	×			×			1.85/2.01
19	7	F	100/80	×			×			2.35/2.34
20	4	F	100/100	×			×			1.96/1.96
21	3	M	90/90	×			×			2.44/2.45
22	5	M	90/100	×						2.80/NA
23	3	M	100/100	×						1.93/NA
24	4	F	100 /100		○	Ⅳ	×			NA/2.00

I_n_ (×), wave I_n_; HJB (○), high jugular bulb; ABR, auditory brainstem response; M, male; F, female; R/l, right ear/left ear; Type, t Manjila and Semaan classification of jugular bulbs.

## Discussion

ABR wave I_n_, the negative peak after peak I, is an electrophysiological phenomenon observed in earlier studies (5, 6). This study identified 24 patients with wave I_n_ by reviewing the ABR results of 221 pediatric patients who were enrolled for cochlear implantation. Interestingly, the HRCT of the temporal bone reveals that 18 of the 24 patients with wave I_n_ demonstrated the presence of HJB (bilateral or unilateral), displaying a high rate of 75% for HJB compared with a rate of 20.8% (41/197) observed in patients without wave I_n_ (ABR). The rate (75%) is also much higher than a rate of 6%–20% for HJB in patients undergoing HRCT of the temporal bone for any reason (8) and a rate of 42% for HJB among 194 children who underwent cranial CT mainly due to head trauma in a recent study (10). By further looking at the JB location, we observed that more than half of the HJBs from patients with wave I_n_ belong to the type 4 JB according to the Manjila and Semaan classification. In a recent study by Hu et al. (12) to screen causative HJB in patients with Meniere’s disease, type 4 JB had a prevalence rate of 8.7% (8/92) in patients with hydropic ears and a prevalence rate of 1.1% (1/90) in patients with non-hydropic ears. Despite the fact that a high rate of type 4 JB was accompanied by wave I_n_ in pediatric patients with profound hearing loss in our study, the physiological correlation between wave I_n_ and HJB remains unclear.

More recently, Kwesi et al. (13) conducted a case–control study to explore the effect of unilateral HJB on hearing loss in 36 patients diagnosed with bilateral LVAS. In addition to the major finding that LVAS with concurrent HJB was associated with higher air conduction thresholds, they also found that the laterality of HJB was mostly in the right ears and that the prevalence of HJB was not correlated with gender and age. In our cohort, significant laterality preference of HJB was not observed (12 right HJBs vs. 14 left HJBs), either the gender difference of patients with HJB (10 males vs. eight females). A proportion (6/24) of the patients in our cohort had a bilateral large vestibular aqueduct; however, this finding was not associated with the presence of HJB.

The present study provokes an interesting discussion regarding the physiological origin of wave I_n_ in the patients. Although ABR wave I_n_ in our study is similar to the summating potential (SP) in electrocochleography (ECochG) described elsewhere (14), there are three main aspects to differentiate wave I_n_ from SP. First, ABR wave I is generated by the distal portion of the auditory nerve (15), whereas SP is a presynapse response, representing direct-current receptor potentials generated by cochlear hair cells. Second, wave I_n_ in the study is a far-field measurement, compared with the SP, which is a near-field recording. In addition, the latency of wave I_n_ was 2 ms in the present study, but the SP showed a latency of approximately 1 ms in previous studies (16, 17). Therefore, wave I_n_ here is unlikely to be a form of SP.

The patients in our study had no residual hearing. However, the possibility of wave I cannot be excluded due to the recorded high thresholds. The recorded wave I could be generated by the residual hair cells in the apical turns of the cochlear triggering the auditory nerves, thus showing a prolonged latency period and a special form. However, the potentials did not yield brainstem neural activation, indicating that the synchronization of potentials is compromised. Alternatively, the recorded waves Ⅰ in the study are synapse potentials, other than neural action potentials.

The strengths of this study include establishing a correlation between ABR wave I_n_ and a high jugular bulb in pediatric patients with profound hearing loss. The limitations of this study include the small sample size of patients with wave I_n_ identified from the cohort and a lack of data on other clinical audiology assessments (such as ECochG) in the cohort due to the retrospective nature of the analysis. Therefore, including additional patients with wave I_n_ in the study group and giving a comprehensive audiology evaluation of the patients would help to further verify the findings in this study.

Collectively, the physiological nature of wave I_n_ needs further investigation. As a high jugular bulb has been implicated in conductive or sensorineural hearing loss in certain patients (18–20), it would be interesting to explore a correlation between a high jugular bulb, altered ABR waves, and hearing loss in future studies.

## Data Availability

The original contributions presented in the study are included in the article/Supplementary Material, further inquiries can be directed to the corresponding author.
